# Lunar cycles and rainy seasons drive growth and reproduction in nummulitid foraminifera, important producers of carbonate buildups

**DOI:** 10.1038/s41598-019-44646-w

**Published:** 2019-06-04

**Authors:** Johann Hohenegger, Shunichi Kinoshita, Antonino Briguglio, Wolfgang Eder, Julia Wöger

**Affiliations:** 1 Institut für Paläontologie, Universität Wien, 1090 Wien, Austria; 2 Department of Geology, Tohoku University, Graduate School of Science, Sendai, Japan; 3 Dipartimento di Scienze della Terra, dell’Ambiente e della Vita, Università degli Studi di Genova, 16132 Genova, Italy

**Keywords:** Palaeontology, Environmental impact

## Abstract

Representatives of the foraminifer *Nummulites* are important in Earth history for timing Cenozoic shallow-water carbonates. Taphonomic complexity explains the construction of carbonate buildups, but reproduction and life span of the constructing individuals are unknown. During the 15-month investigation period, asexually reproduced schizonts and gamonts showed equal proportions in the first half of this period, whereas gamonts predominated in the second half. Oscillations in cell growth are mainly caused by light intensities during chamber construction when minor differences in water depth increase the photosynthetic rate of endosymbiotic diatoms during neap tides. The continuous reproduction rate of *N*. *venosus* throughout the year is increased in subtropical calms by higher summer temperatures and the marginal input of inorganic nutrients during rainy seasons. The expected life span of both gamonts and schizonts are 18 months.

## Introduction

**Figure 1:**
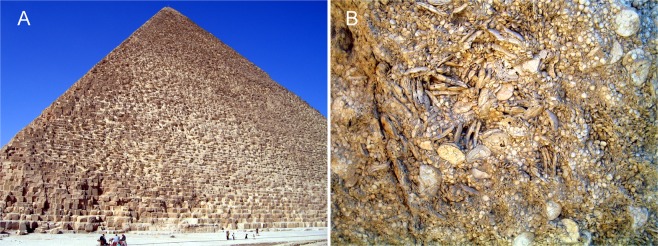
(**A**) The pyramid of pharaoh Chufu (Cheops). (**B**) Surface of a block from the Cheops pyramid showing the composition by *Nummulites ghizehensis* tests with 30 mm large B-generation tests and abundant 5 mm A-generation tests.

Larger symbiont-bearing benthic foraminifera (LBF) originate from different phylogenetic lines and surpass the ‘normal’ test size (<2 mm) of smaller benthic and planktonic foraminifera, with sizes of up to 13 cm in extant^1^ and 19 cm in fossil LBF^2^. They inhabit tropical to warm-temperate shallow seas and house symbiotic microalgae^3,4^ together with bacteria^5^, therefore being restricted to the photic zone. Due to their long history starting in the late Carboniferous at ~ 330 million years before present^6^ they are an informative group commonly used in Earth science either for their biostratigraphic potential in measuring geological time^7,8^ or as paleoceanographic indicators^9–11^. Especially in the Paleogene, members of the genus *Nummulites* are responsible for large carbonate buildups^12,13^ that become important oil reservoirs, aquafers and are used as building materials (Fig. 1).

Although the genesis of ‘nummulite banks’ is intensively discussed^14,15^, the timing of reproduction, growth and longevity of *Nummulites* species and their dependence on environmental conditions are poorly known for the fossil counterparts.

Similarly to cephalopods, foraminifera build their tests by adding a new chamber at every growth step. The ontogeny of the foraminiferal cell can thus be investigated by chamber volumes because the cytoplasm fills the test lumen completely during undisturbed life conditions^16^. This enables observing periodic or instantaneous events recorded by the cell during ontogeny in both extant and extinct species^17^. This opportunity, coupled with the possibility to quantify three-dimensional measurements using computed tomography (CT), yields reliable results^18,19^.

LBF have the advantage of possessing tests with large chamber numbers that are constructed over a much longer time span compared to smaller benthic and planktonic foraminifera. This permits observing the longest and most accurate archive of environmental variations within the life span of a single-celled organism: more than one year at least, reaching several years in living nummulitids^18^.

The growth of foraminiferal cells belonging to Globothalamea^20^ can be modelled by constrained growth functions^16^. Accordingly, the timing of reproduction and longevity of the single specimen can be estimated. Instant variations and constant oscillations around these growth functions have been interpreted as localized stress and environmental oscillations, respectively^16^.

In this respect, only few studies have shown how quantitative biometry can yield precise data on past climatic and environmental changes during the life of LBF^21^. One fascinating result involved determining the influence of tidal and lunar cycles by estimating oscillations in chamber sizes throughout the life span of these LBF based on only a few specimens^18,19^. The currently available data based on chamber number or diameter for estimating life span point to very broad and confusing estimations that range for the very large fossil forms from 100 years^22^ to a few years^23^. Sporadic attempts were made to measure growth of such forms by observations over short intervals^24,25^, but all were done on cultured material and this growth cannot be considered as natural^16,19^.

We therefore conducted a 15-month sampling campaign starting in April 2014. The species *N*. *venosus*, *Heterostegina depressa* and *Operculina elegans*/*complanata* were collected monthly to record their growth and to determine whether they signalize the contemporaneously measured environmental factors.

Using the natural laboratory approach^26^, first results on growth and reproduction based on chamber number and the largest diameter were published^27,28^. The present work presents and discusses cell volume growth of *N*. *venosus* that could explain the influence of environmental factors for the fossil counterparts.

## Collection of Specimens

**Figure 2:**
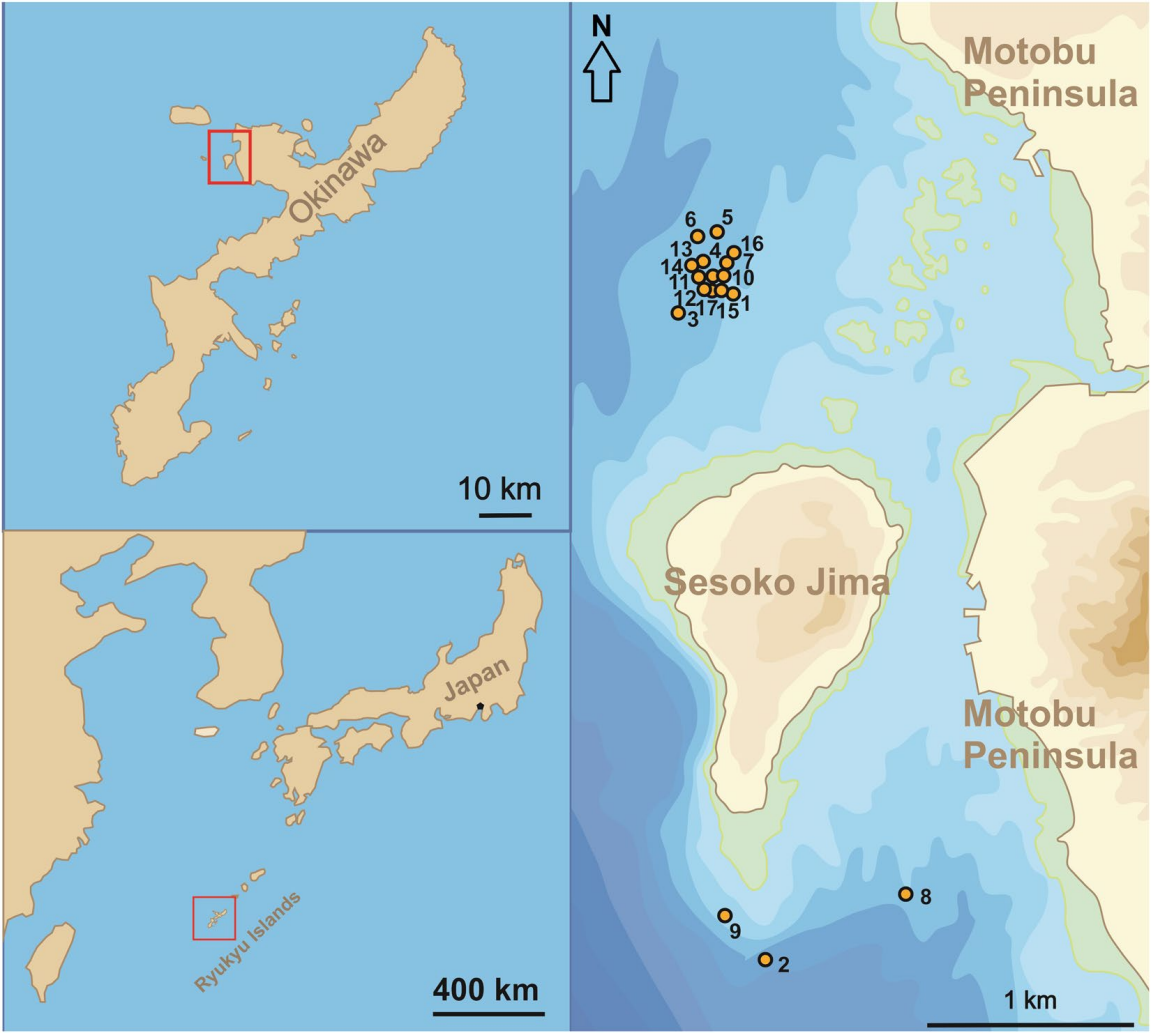
Location of sampling area and sampling sites (sample numbers in Table 1).

The investigation area encompasses the shallow sublittoral around Sesoko Jima (Motobu, Kunigami District, Okinawa, Japan)^27,29^ (Fig. 2). Sampling was performed by Scuba diving from 23 April 2014 to 14 July 2015 (Table 1), starting with two ‘trial samples’ to optimize and standardize sampling procedure^27,29^. Monthly sampling as specified by the ‘natural laboratory’ approach^26^ could not always be done due to bad weather conditions caused by strong winds from the north during winter and spring, and tropical cyclones in summer and early fall^30^. Sample sites of about 50 m depth, where *N*. *venosus* has its abundance optimum^31^, were located northwest and south of Sesoko Island^27,29^ (Table 1). Northwestern sites that are exposed to the open sea were preferred because of their rich content in LBF^30^, but locations in the south protected from strong winds had to be chosen whenever weather conditions to the northwest impeded sampling. Bottom sediments differ between both sample sites at 50m^27,32^: coarse sand at the northwestern sites^33^ and fine sand to silt at the southern sites. This induced abundance differences in all LBF (*Amphistegina*, *Operculina*, *Baculogypsinoides*, *Heterostegina*) depending on their substrate preference^31^.

**Table 1:** Parameters of sampling stations.

Sample number	Date	Longitude	Latitude	Depth	Temperature	Sediment weight (g)	Number of individuals	
							Agamonts	Megalospheres
1	23.04.2014	127°51.388′	26°40.086′	56.0	22.7	715		25
2	02.05.2014	127°52.243′	26°37.126′	46.0	22.3	382		33
3	09.05.2014	127°51.331′	26°40.036′	50.0	21.8	1183		15
4	30.05.2014	127°51.516′	26°40.220′	54.0	23.3	216		2
5	18.07.2014	127°51.532′	26°40.424′	57.5	23.6	999		31
6	19.08.2014	127°51.467′	26°40.423′	56.0	26.2	349	1	4
7	10.09.2014	127°51.528′	26°40.241′	54.0	27.2	797		14
8	03.10.2014	127°52.262′	26°37.425′	41.0	26.9	1377		35
9	10.11.2014	127°51.463′	26°37.351′	41.0	24.7	1573		10
10	11.12.2014	127°51.517′	26°40.218′	47.0	23.5	515		12
11	16.01.2015	127°51.510′	26°40.214′	53.7	21.0	309		18
12	13.02.2015	127°51.508′	26°40.171′	57.0	20.1	488	1	8
13	04.03.2015	127°51.473′	26°40.267′	57.0	22.0	1055	1	32
14	15.04.2015	127°51.454′	26°40.236′	58.0	23.5	506		14
15	18.05.2015	127°51.510′	26°40.276′	55.0	22.9	267		14
16	11.06.2015	127°51.620′	26°40.315′	56.5	24.0	574	2	11
17	14.07.2015	127°51.514′	26°40.160′	50.0	27.4	229	1	6

To prevent error due to clumped distributions, four samples with a minimum distance of 4 m apart were taken with a plastic box at every site. After resting for 24 hours in the laboratory in large trays filled with seawater, living LBF were identified based on complete coloring by their symbiotic microalgae, put into smaller boxes and identified to species level. After separating a few specimens for growth investigations under laboratory conditions^29^, the remaining tests were washed in freshwater and dried. Sediments were rinsed, dried and weighed to estimate species densities^26,27,29^ (Table 1).

## Measuring Techniques

Temperature and photosynthetically active radiation (PAR) were measured at every sampling station in five meter increments using a WTW Multi350i, combined with a depth conductivity cell TA197-LF and a LI-COR LI-250A light meter with a LI-192 underwater Quantum Sensor (Supplements 1, 2). Sampling time was always around 11:00 a.m. to ensure comparability of the transparency measurements. Only sunny days were used in the statistical analyses of PAR.

A total of 270 living *N*. *venosus* were collected. The megalopheric generation (264 individuals) dominated over the rare agamonts (Table 1). Because megalospheres and agamonts differ in growth rates and longevities, only the numerous megalospheres were investigated.

**Figure 3:**
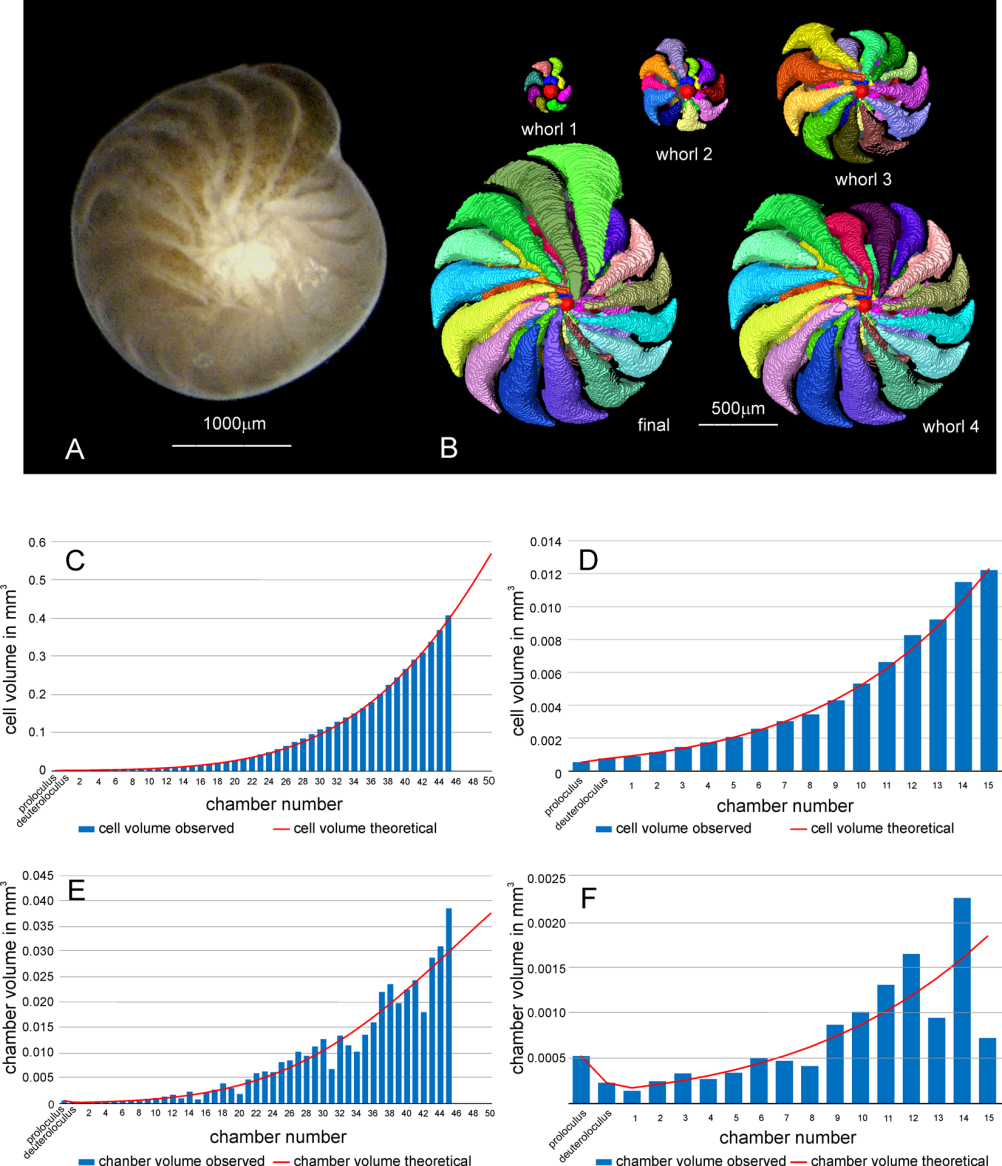
(**A**) Living *Nummulites venosus*. (**B** to **F**) Volume growth of individual N10A_06 (collection date 11.12.2014) and fit by Gompertz function. (**B**) Virtual chamber volumes based on MicroCT micrographs. (**C**) Growth of cell volume; (**D**) Initial cell volume growth; (**E**) Growth of chamber volumes; (**F**) Initial growth of chamber volumes.

To determine cell growth, volumes of chamber lumina were obtained using X-ray tomography^34^. Computer-based algorithms for reconstruction, slicing and 3-D rendering^35^ enabled the virtual reconstruction of chamber volumes (Fig. 3B). Chamber volume measurements are documented in Supplement 3.

## Statistical Methods

**Figure 4:**
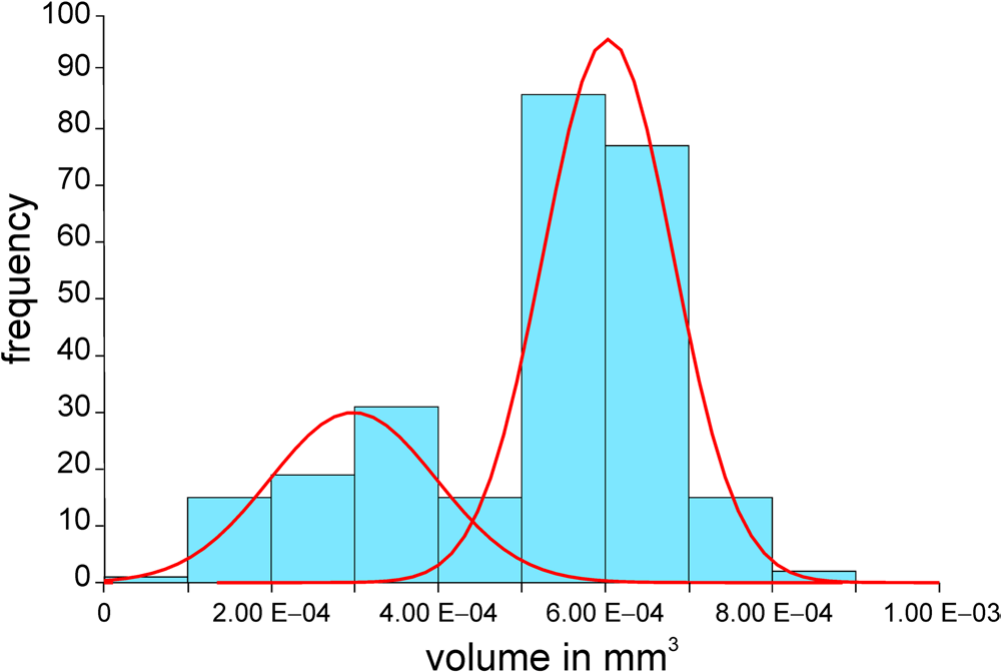
Decomposition of protoconch volumes into normally-distributed components.

Bimodality in protoconch size was tested by decomposition of frequency distributions into normally distributed components finding the cutting point (Fig. 4; Supplement 3). The volume of the foraminiferal cell can be estimated in multi-chambered individuals by the sum of chamber lumens. Cell volume growth was described in two steps. First, cell volumes are related to chamber numbers, showing cell growth in relation to increasing chamber numbers. Second, the timing of chamber construction enables estimating chamber building rates^16^. Combining both functions yields a mathematical model for the observed time-adjusted growth of the foraminiferal cell.

Several mathematical functions can be used for fitting growth in organisms^16,21^. Studies on hyaline larger benthic foraminifera showed that cell growth is limited, which can be fitted by sigmoidal growth functions^16^. Inflection points of sigmoidal functions document the chamber number at the time of asexual or sexual reproduction, i.e. at the end of the individual’s life (semelparity). Chamber volumes, modeled as the first derivative of the cell growth function, decrease continuously during undisturbed growth after the inflection point^16^.

For *N*. *venosus* and *H*. *depressa*, the best fit of cell growth is documented by the Gompertz and Richards’s functions^16^. Here, we used the Gompertz function starting with the first chamber after the nepiont (=embryonal apparatus consisting of a protoconch and deuteroconch). This is based on laboratory observations, where − similar to *Heterostegina depressa*^36^ − the numerous two-chambered offspring are released from the mother’s cell plasm and the following chamber is built within 12 hours^25^.

The statistical estimation of growth functions in foraminiferal tests is strongly influenced by large deviations in final chambers, leading to underestimation of initial chamber volumes. Therefore, chamber volumes were transformed by roots before estimating growth functions, then retransformed. Comparing roots by the AIC (Akaike Information Criterion), the 6^th^ root of chamber volumes led to the best fit by Gompertz functions, preventing overfitting.

Chamber volumes are the basic measurements (Supplement 2). Potential weak resolution of microCT-graphs or strong irregularities in chamber growth impede the estimation of cell growth based on the accumulation of chamber volumes. This requires their complete measurement. Accordingly, the first derivative of the Gompertz function^37^ was used for statistical estimation of the function constants. Functions can be estimated based on a reduced number of chambers, where irregular or not recognizable chamber volumes can be omitted. Specimen N10A_06 serves as an example (Fig. 3C to F).

The precise fit of observed cell and chamber volumes using the retransformed Gompertz function is documented for all individuals in Supplement 4. Calculating an averaged inflection point is important for estimating the life expectancy of *N*. *venosus*. For this purpose specimens with >44 chambers were investigated because fewer chambers lead to overestimation of the upper limit in Gompertz functions (Supplement 4).

The estimation of the mean chamber building rate is based on the ‘natural laboratory’ approach, where frequency distributions of specimens sampled in approximately equal intervals enables estimating the functions describing the increase of chamber numbers through time^26^. Eight samples with a sufficient number of specimens for frequency analyses were distributed over the investigation period^27^ (Table 2).

**Table 2:** Chamber numbers: Parameters of normally distributed components after decomposition of frequency distribution.

day	mean					standard deviation				
	background	generation 1	generation 2	generation 3	generation 4	background	generation 1	generation 2	generation 3	generation 4
09.05.14	27.06	56.24				3.20	5.92			
18.07.14			33.81					8.26		
10.09.14	21.00		45.00			3.17		7.93		
03.10.14	27.21	66.76	49.83			4.24	4,16	6.04		
11.12.14			51.15	26.78				6.53	5.73	
16.01.15	21.00		55.23	39.05		3.17		4.34	7.38	
04.03.15	21.00		56.22	39.25		2.33		5.44	5.58	
11.06.15	24.00			57.00	34.33	2.81			2.86	5.43

First, the frequency distributions of samples must be checked for uni- or multimodality using the Chi-Square test^27^. In case of significant multi-modality, the distributions were decomposed into normally distributed components using nonlinear regression based on numerical mathematics. To estimate the mean chamber building rate, means and standard deviations of each component in the samples were used^26,27^. To estimate the chamber building of each component at the sampling date, the maximum chamber numbers and their normalized standard deviations based on the averaged coefficient of variation were calculated^26^ (Table 2).

The best fit for the increase in chamber numbers of the components, especially in the initial part, is provided by the Michaelis-Menten function^16,37^. The inverse function enables estimating the time interval between the reproduction date and construction of the chamber. Therefore, the age of every specimen can be determined using its final chamber number.

**Figure 5:**
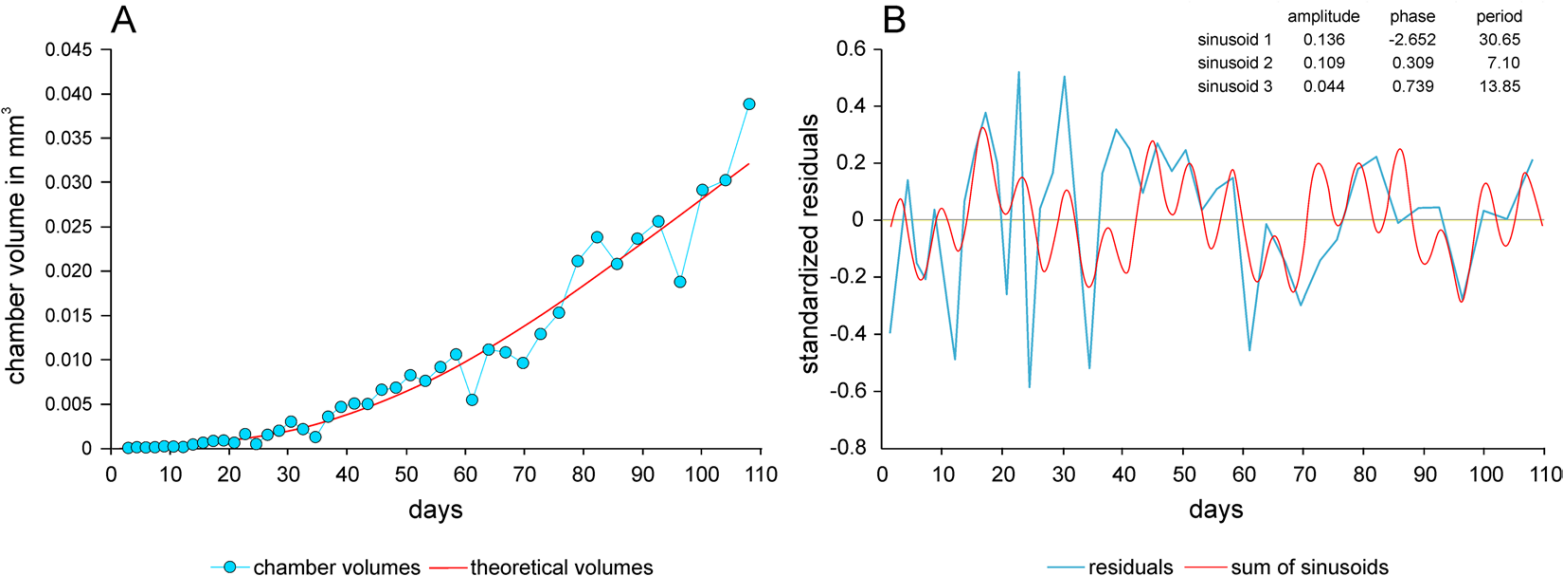
Chamber volume growth of individual N10A_06 related to time. (**A**) Fit by time-adjusted Gompertz function. (**B**) Standardized residuals to the growth function and fit of periods by sum of sinusoids based on Nyquist frequencies.

Combining chamber volume growth based on chamber numbers with the chamber building function yields the time-dependent growth function for chamber volumes. For constants of the Michaelis-Menten function, common values for all components can be used^16,26,27^. Specimen N10A_06 serves as an example (Fig. 5A).

Deviations of observed chamber volumes from expected values based on the adjusted time-dependent growth function were calculated using standardized residuals^16,26,27^. The observed values oscillate in all specimens around the expected values given by the growth function. This is exemplified by specimen N10A_06 in Fig. 5B and documented for all specimens in Supplement 5.

Oscillations around the theoretical growth function were fitted by sums of functions based on sinusoidal regression using Nyquist frequencies^38,39^ (Fig. 5B). Fittings of all individuals are documented in Supplement 5. Periods of significant sinusoids in every individual were summed, yielding 809 periods. They were decomposed into normal-distributed components determining the main periods^18,19^.

Based on sums of sinusoids, the reproduction day and the day of constructing the last chamber can be estimated for every specimen using cross correlation between the tidal calendar and the sum of sinusoidal function. Starting with the sampling date, backwards correlation in Lags^40^ was done for every specimen until the date that equals the time interval between the construction of the last and the preceding chamber.

Correlations between water transparency and reproduction dates were based on two measurements of transparency. PAR was measured on the sampling day around 11:00 in 5 meter intervals down to 50 m depth (Supplement 1). The instantaneous measures of PAR at 50 m depth used for correlations depend strongly on weather conditions and season. Accordingly, the exponent *AC* (Attenuation Coefficient) of the depth function for PAR^30^ at each sampling date was used as a measure independent of the daily weather conditions. This ensures the comparability of all sampling sites.

All complex analyses were calculated with the program packages IBM SPSS and PAST 3.2, whereas the program Microsoft Excel was used for simpler analyses.

## Results

Based on protoconch volumes, the megalospheric generation of *N*. *venosus* can be divided into two groups (${\mu }_{1}$ = 2.98 E-04 mm^3^, ${\sigma }_{1}$ = 9.99 E-05; ${\mu }_{2}$ = 6.03 E-04 mm^3^, ${\sigma }_{2}$ = 7.74 E-05), whereby the group with larger proloculi dominates (70.7%; Fig. 4). They can be interpreted as representatives of the two generations differing in reproduction^16,17,19,41–43^. The generations interpreted as asexually reproducing schizonts (A1-generation) and sexually reproducing gamonts (A2-generation) differ in their reproduction. Nonetheless, they are similar in test form, size and life expectancy. We therefore treat them as a single generation of megalospheres reproducing at the end of their life (semelparity).

**Figure 6:**
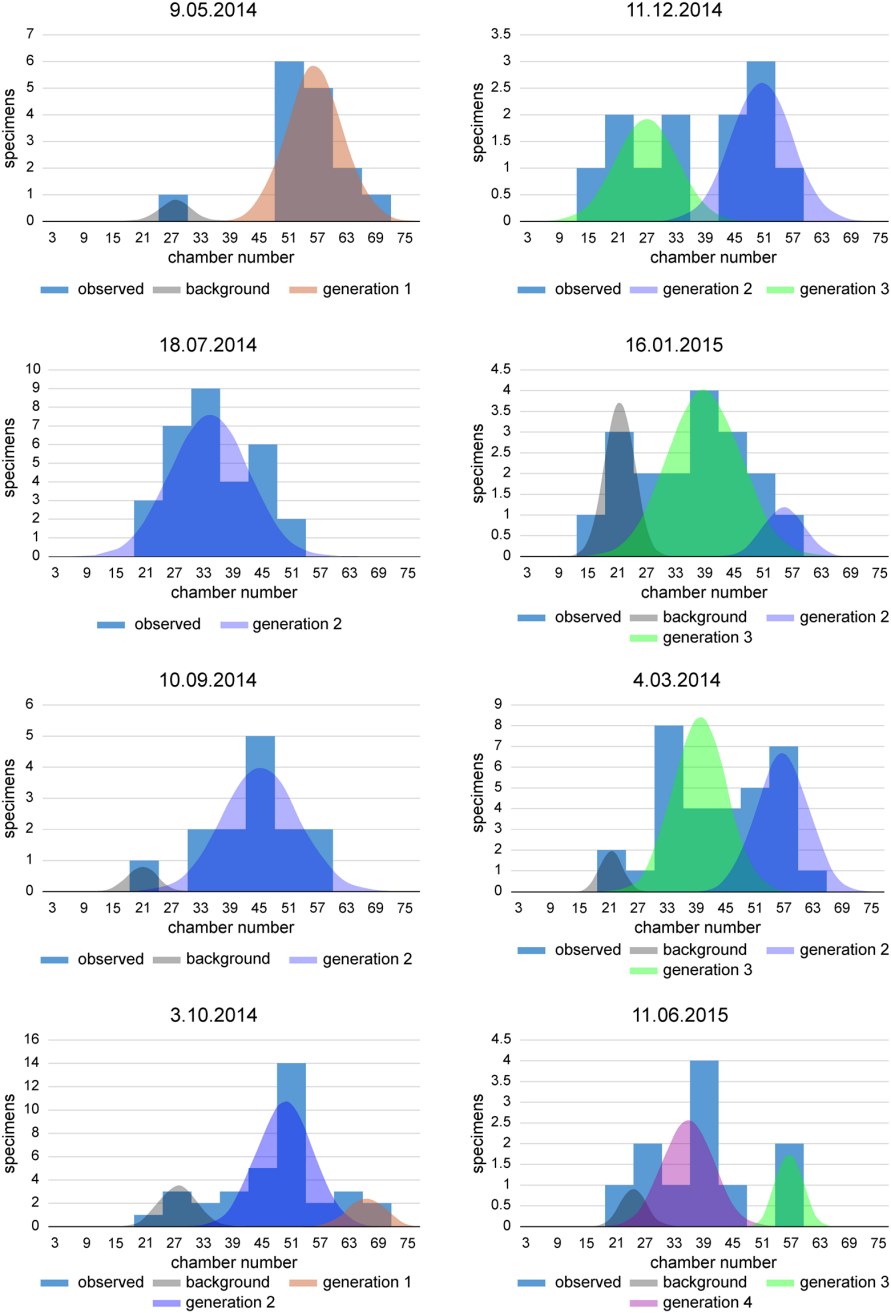
Decomposition of frequency distributions into normally distributed components.

Former investigations demonstrated a continuous reproduction of megalospheres over the investigation period^27^. Beside this background reproduction, the decomposition of sample frequencies into components revealed the presence of four megalospheric generations, showing intervals of intensified reproduction (Table 2, Fig. 6). Initially (9 May 2014), except for the low background reproduction, the dominant megalospheric generation is characterized by a mean of 56.2 chambers. This is attributed to an intensified reproduction interval during the previous months (Table 2). In the following sample (18 July 2014) only one megalospheric generation with a mean of 33.8 chambers is present (Table 2, Fig. 6). This points to a second intensified reproduction interval between the first and the second sampling dates. Due to the low total frequencies, the first megalospheric generation starting with numerous individuals before May is not represented in the July sample, but can be found in the sample from 3 October 2014 exhibiting the highest mean (66.7 chambers) of all megalospheric generations. The second megalospheric generation, starting between May and June, shows a significant trend to higher chamber numbers in the following months (Fig. 5), finally represented on 4 March 2015 by a mean of 56.2 chambers.

A clear bimodal distribution characterizes the 11 December 2014 sample. The generation with the higher chamber numbers follows the trend of the second megalospheric generation. A third megalospheric generation, with frequencies equal to the second megalospheric generation, has a mean of 26.8 chambers including the background reproduction. This hints at a further intensified reproduction interval between late September and December 2014 (Fig. 6). Chamber numbers of the third megalospheric generation increase until the end of the investigation period, finishing with a mean of 57.0 chambers in June 2015. In the January and March 2015 samples, the dominance of the third (younger) megalospheric generation over the second (older) component is characteristic. This follows the rules of population dynamics, with overlapping generations for test size^44–47^ and chamber number^26,27^. The final sample from 11 June 2015 demonstrates the annual trend in population dynamics. Beside the constant background reproduction, a new megalospheric generation (number 4) originating in the contiguous earlier months is slightly separated from the background generation. The third megalospheric generation starting in autumn 2014 becomes less abundant in comparison with the new generation (Fig. 6).

**Figure 7:**
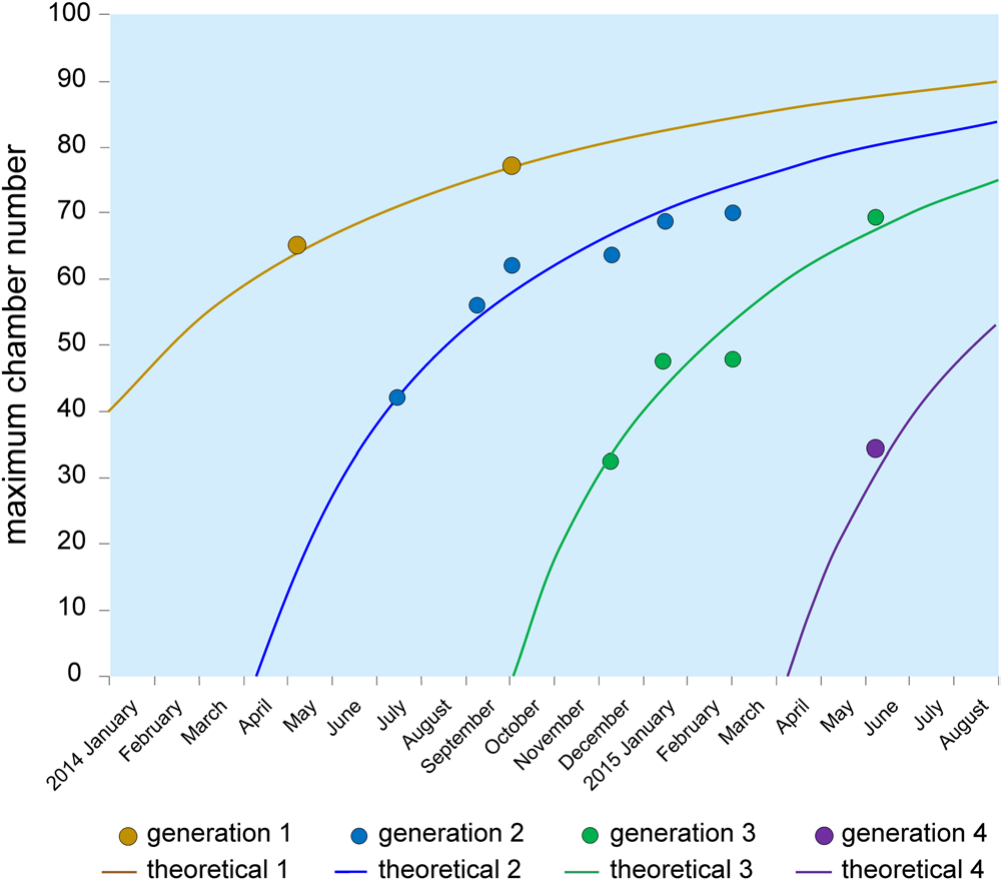
Fit of generations with intensified reproduction by a single Michaelis-Menten function *n* = 109.4 *t*/(154.7 + *t*) with different onset times.

Iterative estimation of constants for the Michaelis-Menten functions using nonlinear regression (SPSS) resulted in constants ${L}_{\infty }=$ 109.42 and *k* = 154.67. This documents a common function for all megalospheric generations (Fig. 7). Based on this calculation, the origin of functions attributed to generations designates intervals with intensified reproduction. Starting dates with intensified reproduction are positioned on 9 April 2014 for the second and 3 October 2014 for the third megalospheric generation. The annual recurrence of megalospheric generations leads to positions on 3 October 2013 for the first and 9 April 2015 for the fourth megalospheric generation (Fig. 7). This is confirmed by the first megalospheric generation on 3 October 2014, exactly at the origin of the third generation (Fig. 7).

The life expectancy of *N*. *venosus* can now be estimated using the inflection point of cell growth inserted into the inverse common Michaelis-Menten function. Estimations using individuals with few chambers often show extreme values (>1000). We therefore used specimens with >44 chambers to estimate a common maximum inflection point. The common inflection point is positioned at 85.2 chambers, but the lowest limit inferred by the subtraction of one standard deviation starts at 68.9 chambers. Using the inverse Michaelis-Menten function for estimating the reproduction time of individuals, the time intervals range from 8 to 21 months with a mean at 18 months.

All specimens show oscillations in chamber volumes around the time-adjusted growth curve (Fig. 5A, Supplement 4). Standardized residuals from the theoretical growth functions were fitted by periodic functions based on sinusoids using Nyquist frequencies (Fig. 5B, Supplement 4). Depending on chamber number, up to 5 significant periods could be detected. In contrast, a maximum of 2 significant periods can be expressed in specimens with <30 chambers.

**Figure 8:**
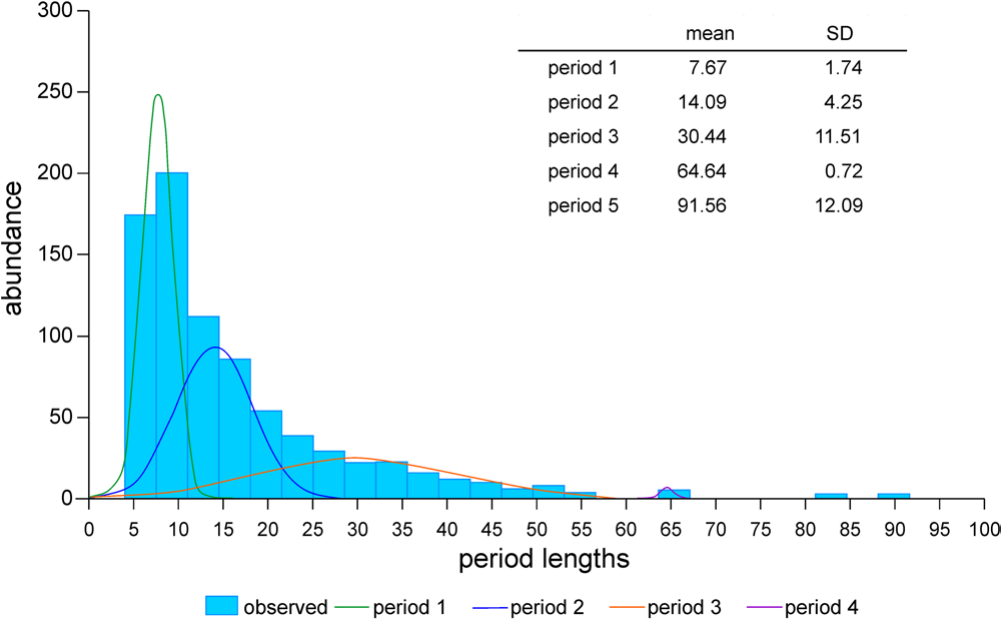
Frequency distribution of significant periods found in all individuals and decomposition into normally distributed components.

In order to check the dominant periods in *N*. *venosus*, the frequency distribution of 810 periods originating from 263 investigated specimens was decomposed into normally distributed components. This yielded five periods with averages of 7.7, 14.1, 30.4, 64.6 and 91.6 days (Fig. 8). They are similar to the periods found in *Heterostegina depressa* from Okinawa and Hawaii^19^, *N*. *venosus* from Okinawa and Belau and *Cycloclypeus carpenteri* from Okinawa^18^. All former investigations are based on a much lower number (maximum 15) of specimens. The exemplary individual N10A_06 shows significant periods close to the general means (Fig. 5B). In this individual, restriction to the first 3 periods is caused by life span, where 45 chambers indicates a lifetime of 108 days. This hinders the detection of larger periods, because periods require lifetimes as long as the cycle needs to repeat itself^18^. Therefore, the minimum chamber numbers for detecting periods are 10 chambers for period 7.7 days, 17 chambers for period 14.1 days, 31 chambers for period 30.4 days, 50 chambers for period 64.6 days and 60 chambers for period 91.6 days.

In the next step, we determined the approximate birth date of specimens and the construction date of their last chamber based on the time-adjusted growth functions and periodic regressions. Because periods around 14 days were represented in almost all specimens, the standardized residuals and their fit with sinusoidal regressions could be cross-correlated with tidal stands at noon (11:00 am), when samples were taken^48^. Starting with the sampling day, the backwards cross-correlation stopped when arriving at the time interval between the construction of the last and the preceding chamber based on the Michaelis-Menten function. These backward intervals (lags) range from −2 days (minimum of 15 chambers) to −7 days (maximum of 60 chambers).

**Figure 9:**
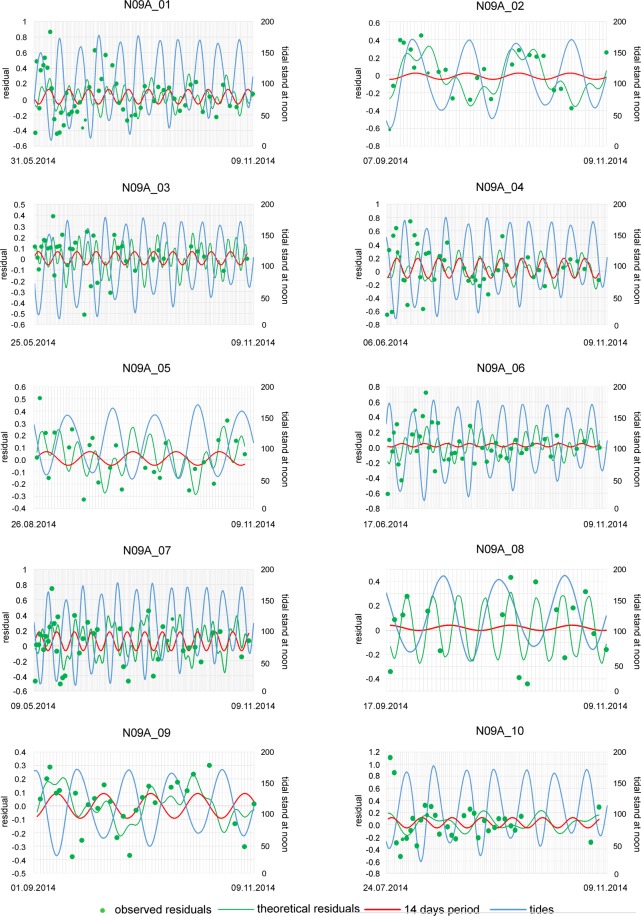
Correlation between tidal stands at noon and periodic chamber volume growth of all individuals sampled on 10.11.2014; birth dates indicated.

Using cross-correlation, almost all specimens demonstrated highest significant negative correlation between the sum of sinusoids and tidal stands. Significant correlations with neap tides are evident especially when regarding the single sinusoid with a period of around 14 days. Results for all specimens are shown in Supplement 5, but also documented for individuals collected on November 10, 2014, where significant correlations with neap tides are expressed especially in specimens with higher chamber numbers (Fig. 9).

**Figure 10:**
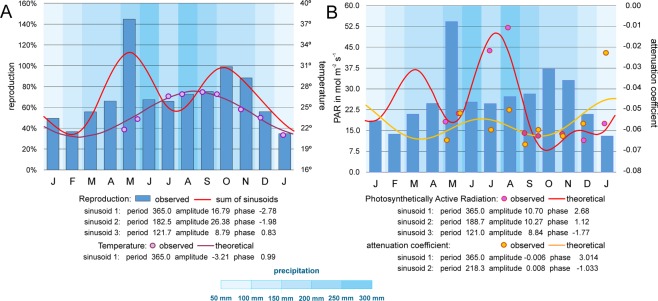
(**A**) Frequency diagram of reproduction events in 2014 and fit by sum of sinusoids based on a harmonic series of periods. Temperature measured at 50 m and fit by a single sinusoid including measurements until 11 June 2015 (Supplement 1). Significance of fit for temperature: ${R}^{2}$ = 0.884, $p({H}_{0})$ = 9.502E-07, (**B**) Frequency diagram of reproduction events (monthly means), PAR at 50 m and attenuation coefficients in 2014. Fit by sums of sinusoids including measurements until 11 June 2015 (Supplement 1). Significance of fit for PAR: ${R}^{2}$ = 0.885, $p({H}_{0})$ = 4.810E-06 and for attenuation coefficients: ${R}^{2}$ = 0.543, $p({H}_{0})$ = 0.022; averaged monthly precipitation measured from 1996 to 2015 (Japan Meteorological Agency^48^).

The knowledge about birth dates enables calculating the frequency distribution of birth dates over the year based on percentages (Fig. 10A). The distribution is characterized by two peaks, the first in March/June and the second in October. Two local minima are positioned in February and July. Again, this is evidence for continuous reproduction over the year with a boost in late spring/early summer and fall. The coincidence of this distribution with rainy seasons is manifested by significant correlation (${R}^{2}=$ 0.318, $p({H}_{0})=$ 0.045) with monthly precipitation averaged over the years 1996 to 2015^48^ (Fig. 10A).

Fitting the frequency distribution of reproduction onsets with the sum of sinusoids following the harmonic series (365 days starting period) resulted in an optimal fit (*R*^2^ = 0.730 with the probability of non-correlation $p({H}_{0})=$ 1.518E-04). This year-by-year repeatable periodic oscillation is composed of 3 sinusoids marking a full and a half-year sinusoid completed by a sinusoid with 4 months period. The summary function is determined by differences in sinusoid phases and to a minor degree in their amplitudes (Fig. 10A).

We tested the correlations of reproduction onsets with the environmental factors precipitation, water temperature and *PAR* (both at 50 m depth) and the attenuation coefficient *AC* using periodic functions of the collected data (Supplement 1). For temperature, starting with a period of 365 days, decomposition resulted in a single, highly significant sinusoid with a minimum of 20.8° on 28 February 2014 and maximum of 27.2° on 28 August 2014 (Fig. 10A).

To obtain trends in PAR measured at 50 m depth, we used only sites with clear days and maximum irradiation at noon. Significant fitting with periodic functions resulted in 3 sinusoids with periods of 365, 188.7 and 121.0 days. This is close to the harmonic sequence of periods with 356, 182.5 and 121.7 days. Starting with 13.6 mol m^−2^ s^−1^ on 1 January 2014, PAR increases to a local maximum of 36.7 mol m^−2^ s^−1^ on 22 March 2014. A local minimum of 18.0 mol m^−2^ s^−1^ on 21 May 2014 is followed by the total maximum of 50.0 mol m^−2^ s^−1^ on 28 July 2014. The strong decrease to the total minimum of 7.7 mol m^−2^ s^−1^ on 18 October 2014 is succeeded by a weak increase, intensified after 1 January 2015 (Fig. 10B).

Transparency measures using the AC are independent of season and weather conditions. These data can be fitted by a periodic function based on the sum of 2 sinusoids (Fig. 10). The weak AC (−0.045) around January, documenting high transparency, is followed by a drop to −0.064 in March/April. This period of low transparency is succeeded by an increase in early summer (−0.055) with a subsequent decrease to −0.062 in early fall (Fig. 10B).

The influence of transparency and temperature on reproduction was tested by correlation analyses (Table 3). Temperature at 50 m depth correlates positively with reproduction, whereby the temperature peak on 28 August 2014 (Fig. 10A) is slightly different from the reproduction peak of the sinusoid with the 365-day period positioned on 24 July 2014 (Fig. 10A). This is also expressed by different phases in both sinusoids (phase_reproduction_ = −0.012, phase_temperature_ = 0.99).

**Table 3:** Pearson’s correlations between reproduction, precipitation, temperature, PAR at 50 m depth and transparency (AC). Lower triangle: correlation coefficients, upper triangle: p(H_0_) of non-correlation.

reproduction	*observed*	*theoretical*	precipitation	temperature	PAR (50 m)	AC
*observed*		*0*.*0002*	*0*.*0449*	*0*.*1644*	*0*.*4739*	*0*.*1364*
*theoretical*	**0**.**8536**		*0*.*0154*	*0*.*0985*	*0*.*3879*	*0*.*1077*
precipitation	**0**.**5636**	**0**.**6537**		*0*.*0029*	*0*.*2937*	*0*.*1787*
temperature	0.4097	0.4780	**0**.**7545**		*0*.*7272*	*0*.*5362*
PAR (50 m)	−0.2182	−0.2616	0.3155	0.1073		*0*.*4222*
AC	−0.4360	−0.4669	−0.3974	−0.1890	−0.2438	

The negative correlation between reproduction and PAR at 50 m depth (Table 3) demonstrates the influence of transparency (Fig. 10B). The peak in March is consistent with weak reproduction in late winter/early spring. The intense decrease of PAR in May is coupled with highest reproduction, followed in July by the opposite relation of high transparency versus low reproduction. The subsequent decrease to a minimum in October is congruent with an increase in reproduction, becoming a local maximum. Afterwards, the slow increase in PAR is concurrent with a slow decrease in reproduction. These tendencies are intensified from January until March (Fig. 10B).

The negative correlation between reproduction dates and AC (Table 3) roughly confirms the results obtained by the instantaneous PAR measurements at 50 m depth. High transparencies correlate with low reproduction in winter (AC: −0.045) and summer (AC: −0.055), while weak transparencies in spring (AC: −0.064) and fall (AC: −0.062) are correlated with intensified reproduction (Fig. 10B).

**Figure 11:**
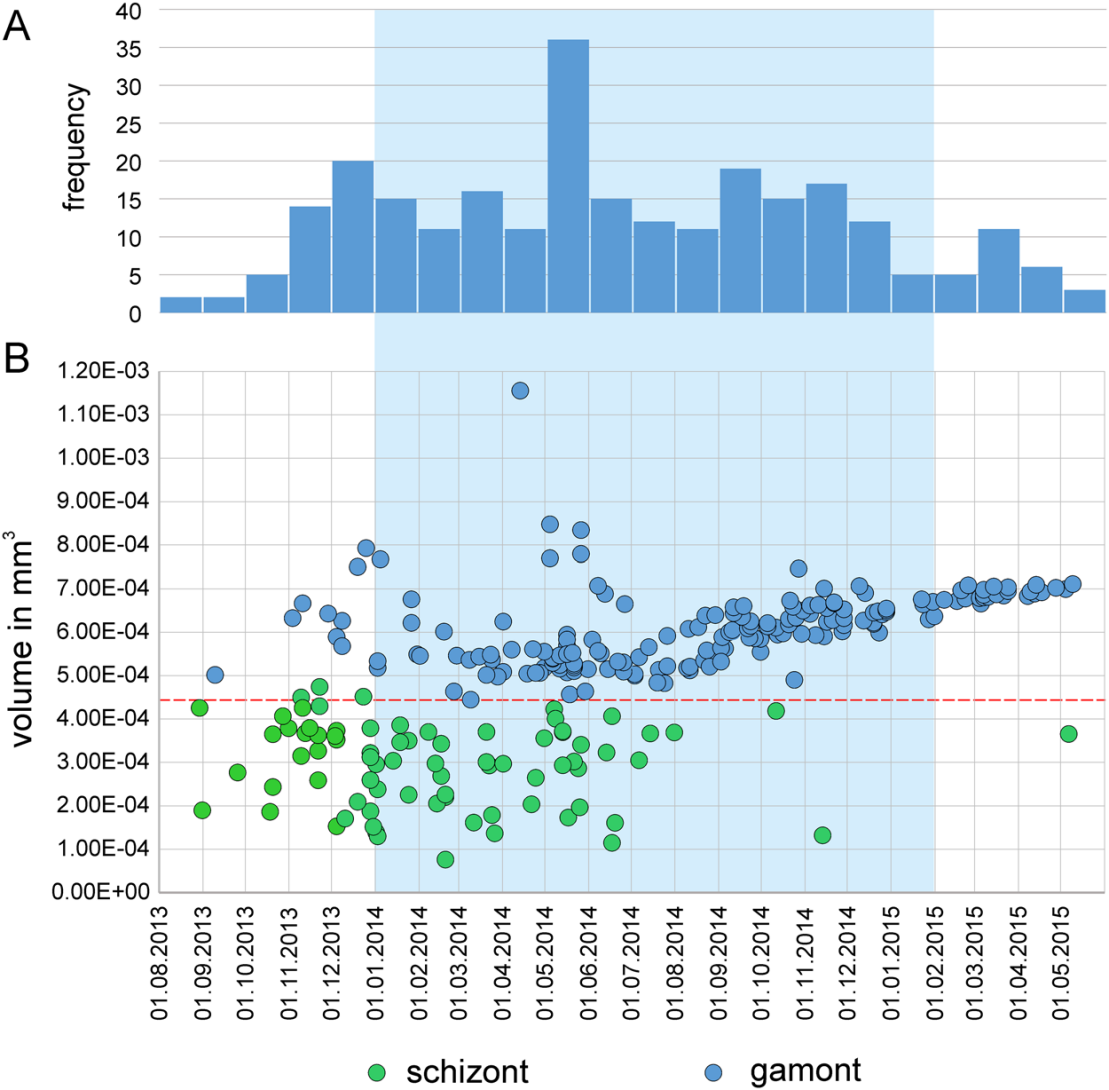
Protoconch volumes at reproduction dates; (**A**) Monthly frequencies; (**B**) Volumes; red line differentiates between schizonts and gamonts (Fig. 4; Supplement 3).

The knowledge about reproduction onsets enables differentiating schizonts (with smaller proloculi) from gamonts (with larger proloculi) in the period between August 2013 and July 2015 (Fig. 11). Here, the different numbers of individuals within samples leads to non-normalized frequencies, but values are similar to onset frequencies standardized by percentages (Fig. 10A). These unweighted frequencies again document the continuous reproduction during the year with peaks in May 2014 and in September to November 2014. Moreover, the peak in winter 2013/2014 confirms former observations^27^ (Fig. 11A). The differences in both generations is remarkable. While from September 2013 until March 2014 the smaller proloculi, interpreted as schizonts, were fractionally more abundant, the generation with larger proloculi (gamonts) became more abundant between April and October 2014 and dominated almost completely in the following months, showing significantly increasing volumes (Fig. 11B).

## Discussion

During the investigation period, only a few agamonts of *N*. *venosus* were found (Table 1). Nonetheless, their growth based on chamber volumes could be measured, enabling comparison with megalospheres, although statistical investigation of reproduction events and life span using the ‘natural laboratory’ approach^26^ was not possible.

The numerous megalospheres (separated according to protoconch size into asexually reproducing schizonts and sexually reproducing gamonts; Fig. 4) were successfully investigated with respect to cell growth, life expectancy and reproduction events because their test construction is equivalent.

Undisturbed cell growth in gamonts/schizonts can be modelled by limited growth functions. The used Gompertz function starts after the nepiont stage (consisting of a protoconch and deuteroconch). These first two chambers differ in form and size from the following ones, which are arcuate and embrace the former test laterally towards the umbilicus, leading to involute tests. The maximum distances of the chambers’ outline to the test center (‘Marginal Radius Vectors Length’^49^) increase exponentially, leading to logarithmic spirals (Fig. 3A). Modelling these spirals by mathematical functions^49,50^ helps checking the constancy of test form within a species and differences to other species, ecophenotypes or generations^18,19^. These spirals are apparently genetically controlled because, after disturbance of cell growth, all specimens try to attain the characteristic spiral form of the species (or ecophenotype, generation) as soon as possible^16^. Reduced chamber volumes induced by weak disturbance (e.g., starvation) are compensated by reducing septal distances and thus retaining the spiral outline^17^. Repair mechanisms after stronger disturbance such as fractioning also help attain the ‘normal’ test form in many nummulitid foraminifera, but often via a large number of ‘deformed’ repair chambers^16,51^.

In contrast to test outlines that follow an unlimited exponential growth, cell growth in *N*. *venosus* can be modelled by the limited Gompertz function. The inflection points of this function highlight the termination of cell growth, at which time sexual or asexual reproduction occurs^16^. The inverse Michaelis-Menten function for estimating the life span of individuals, which is capped by reproduction, yields values ranging from approximately 8 to 21 months. This result supports previous estimations of 1.5 years obtained from laboratory cultures^36^ and 432 days from a previous estimation using chamber numbers of the data set presented here, based on the maximum observed life span of investigated specimens^27^.

Regarding the standardized oscillations around the time-adjusted chamber-growth function, the significant periods (with averages of 7.7, 14.1, 30.4, 64.6 and 91.6 days) can be equated with weekly, spring-neap tide, lunar, bimonthly and seasonal cycle periods. Similar cycles have been documented in the nummulitids *N*. *venosus*, *Heterostegina depressa* and *Cycloclypeus carpenteri*^18,19^. The weekly, spring-neap tide and lunar cycles were documented in all specimens with >30 chambers, but the larger cycles could be represented only in specimens with >50 chambers (bimonthly periods) and >60 chambers (seasonal periods), explaining their low abundance (Fig. 8). Examined in more detail, the phases of weekly and spring-neap tide cycles are very similar (Fig. 5B). Accordingly, weekly cycles influence spring-neap tidal cycles (Fig. 9).

The strong correlations between lunar cycles and chamber building must involve factors depending on lunar cycles that influence cell growth. Enhanced growth in symbiont-bearing benthic nummulitids could be caused by the better availability of organic carbon produced by the endosymbiotic diatoms^52,53^, which belong to a monophyletic group of *Thalassionema*-like Fragilariophyceae^54^. Because nummulitid foraminifera rarely take up organic food from outside (e.g., bacteria), they depend to a high degree on the organic carbon produced by the symbiotic microalgae. Accordingly, they act as mixotrophic to approximately autotrophic organisms^55^. In this case, they must provide their symbionts with inorganic nutrients obtained by incorporated bacteria^5,56,57^, as waste products of host metabolism^55^ or taken up through seawater vacuoles. The latter mode is also important for the calcification of the chamber wall^58–60^.

Growth studies on living LBF demonstrate the positive dependence on light^55,61–63^, while the influence of inorganic nutrients seems to depend on their concentration, leading to positive effects at low input^3,55,62^ and negative effects at higher input^3,62–64^. Very limited knowledge is available on the effect of the bacterial community hosted within the foraminifera cell^5,57^. This community may play an important role in foraminiferal physiology. A related hypothesis on nutrients states that overabundant inorganic nutrients suppress foraminiferal growth because they induce higher growth rates in the symbionts. Releasing the symbionts from nutrient limitation may involve a switch from low symbiont growth and high translation of organic carbon to high growth rates of symbionts coupled with low organic carbon transfer to the host^4,65^.

**Figure 12:**
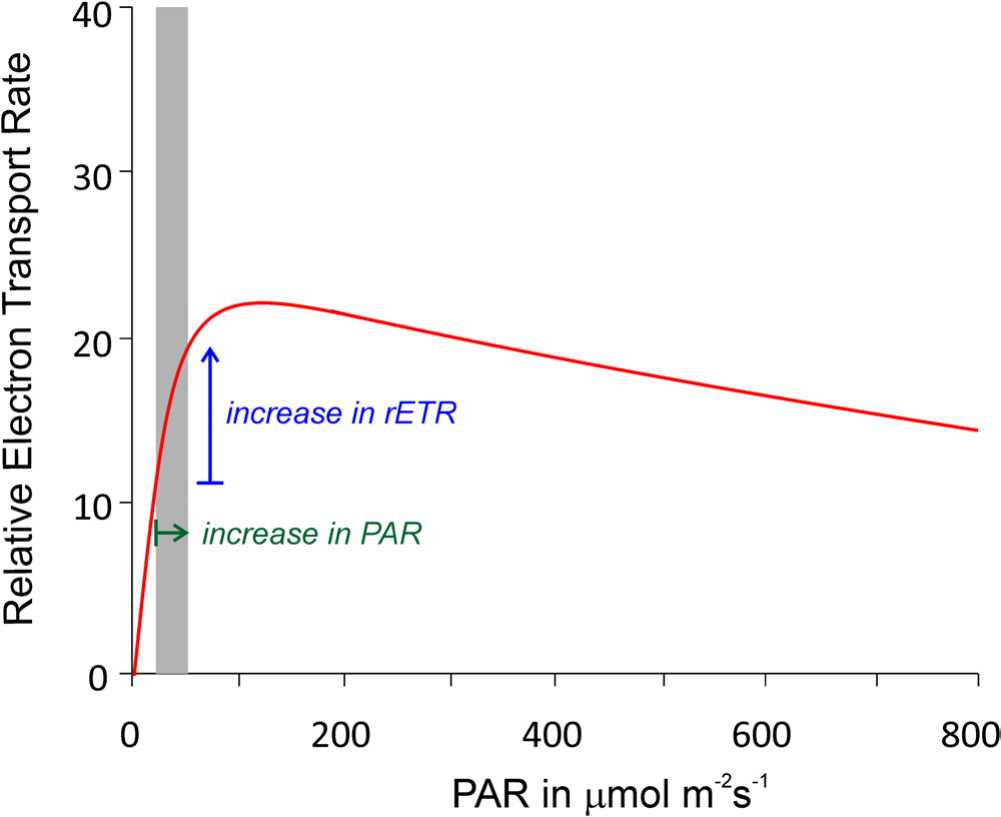
Light curves for diatom symbionts in *Heterostegina depressa*^66^ and the effect of increasing PAR at 50 m water depth during neap tides expressed in the relative Electron Transport Rate.

The positive correlation to neap tides with marginally lower water columns could be effected by an increase of PAR. The decline of sea level by 1.5 meters during neap tides as experienced in the investigation area^48^ leads to an increase between 5 and 20 PAR, whereby light intensity depends on the one hand on transparency (AC) and, on the other, on insolation at the water-surface (the latter changing seasonally). Since endosymbiotic diatoms achieve their optimum photosynthetic rate at minor irradiation around 125 PAR^66,67^, the small increase during neap tides starting from a mean of 22.5 PAR at 50 m (with an AC of 0.4, Supplement 1) may promote the production of organic carbon that could be offered to the host (Fig. 12)

Nutrient dynamics dependent on tides are important in the littoral and uppermost sublittoral within humid climate zones characterized by strong freshwater input. Especially in the littoral and uppermost sublittoral, the input of inorganic nutrients via rivers^68^ or groundwater^69^ is higher during neap than during spring tides. The limitation of nummulitids to oligotrophic environments that are rare in inorganic nutrients originating from runoffs makes the influence of neap tides on foraminiferal growth – whether positive or negative – less probable.

Finally, bottom currents during neap tides are weaker than during spring tides, potentially helping to stabilize the hydrodynamic conditions during the sensitive process of calcification, where minor disturbances can lead to test breakage or chamber malformation.

Ongoing reproduction of *N*. *venosus* during 2014 peaked in May/June and September/October, which clearly corresponds with the rainy seasons averaged over 20 years (Table 3, Fig. 10). The averaged course of reproduction correlates with temperature, showing a maximum in August and a minimum in February (Table 3, Fig. 10). Lower reproduction in January/February and July/August is coupled with high water transparencies, both documented by the instantaneous measurements of PAR at 50 m depth measured at noon on full sunshine days and by the attenuation coefficients AC (Table 3, Fig. 10B). This negative correlation between PAR and reproduction cannot be traced back to a positive influence of light. On the contrary, the weakly increased input of inorganic nutrients by runoffs during the rainy season, documented by low transparencies (Fig. 10B), possibly raised the reproduction rate.

Separating individuals by protoconch size into schizonts and gamonts provides additional insights (Fig. 4). Both generations reproduced asexually, schizonts by mitosis and gamonts by meiosis. Their proportions change over the year (Fig. 11B). Schizonts dominate from November 2013 until February 2014, becoming equal in proportion with gamonts from March 2014 to June 2014. A strong dominance of gamonts starts in August 2014, where protoconch size significantly increases until the end of the investigation period (Fig. 11B). Explaining this (non-periodic) trend within the investigation time is difficult. One possibility is that longer-lasting changes in environmental conditions may alter reproduction strategies, especially at water depths that are sensitive to hydrodynamics. A similar shift from dominant schizonts to dominant gamonts occurs in *Heterostegina depressa* between 30 and 60 m depth^43^. In shallower parts, strong hydrodynamics hinder gametogamy that would establish a sexual reproduction cycle^70^, while gametogamy dominates below 60 m. Accordingly, even slight long-term changes of hydrodynamics in the transition zone may explain the alternations in reproduction strategies. Finally, the birth date, life span and asexual reproduction of agamonts play an important role for the offspring of gamonts, predominating in the second part of the investigation period. The low individual numbers examined here warrant further investigation of population dynamics.

The following factors characterize life in *N*. *venosus* and support its choice as a model for fossil and extant nummulitid foraminifera. While individual growth depends on light, sexual and asexual reproduction are determined by increased inputs of inorganic nutrients, especially during the rainy season. Excessive inorganic nutrients, however, repress both growth and reproduction.

## Conclusion

The biology and physiology of modern LBF allow inferences on fossil forms. *Nummulites venosus* represents the single extant representative of the genus, which includes the most important species for carbonate buildups in the Paleogene. Each foraminiferal chamber is a precious archive of the environmental conditions at the time of chamber formation and can be used to obtain information about environmental trends. The clear correlation between growth and lunar cycles opens the possibility to observe past variations in fossil *Nummulites* and *Assilina*, including the large multispiral agamonts^16,23^.

The major results achieved in this study are that undisturbed cell growth primarily depends on light intensities in relation to the optimum photosynthetic rate of the endosymbiotic microalgae. For nummulitids inhabiting the mid and deep euphotic zone, minor differences in depth caused by variable tidal heights enhances the photosynthetic rate of the endosymbiotic diatoms during neap tides because these microalgae achieve their optimum photosynthetic rate at low PAR. Seasonal changes in light intensities are more efficient at higher latitudes. Accordingly, the above dependencies enable approximating the geographic position of the fossil habitat based on differences in cell growth rates^19^. A further effect of neap tides could be facilitated chamber construction due to weaker bottom currents.

Rainy seasons (in combination with temperature) stimulate reproduction in *N*. *venosus* by an increased input of inorganic nutrients, making reproduction dependent on latitude. In the equatorial region, constant reproduction rates occur throughout the year, whereas reproduction is intensified in subtropical calms during rainy seasons. In arid climates, however, temperature seems to be the single factor inducing intensified reproduction during summer months.

Our understanding of the ratio between asexually reproduced gamonts and schizonts in the fossil record is still poor, but it is clear that schizonts are abundant and should not be neglected. This can have major consequences in the definition of biozones in geological history, because most Cenozoic LBF biozones are defined by species whose diagnostic characters are based on biometric parameters involving nepiont (protoconch and deuteroconch) measurements of the megalospheres. Regarding the populations as a mixture of both generations will result in revised and improved biozone boundaries.

Additional questions about growth and reproduction in fossil nummulitids remain to be answered, especially the growth, life span and reproduction of the large agamonts (B-generation). These aspects are important for the number of schizonts produced by agamonts (approximately 1800 schizonts or gamonts per agamont) in relation to the number of schizonts produced by the preceding schizont generation.

In summary, growth in nummulitids is affected by lunar cycles, and reproduction is driven by seasonal oscillations. Whilst such effects are difficult to detect and interpret for fossil representatives, they are crucial in tracking down the signal of climatic deterioration in short-term studies. We show that concentrating the effort on extant taxa is the only path to gain a clear understanding of major evolutionary trends in geological history.

## Supplementary information

Dataset 1

Dataset 2

Dataset 3

Dataset 4

Dataset 5
